# *Hmg1* Gene Mutation Prevalence in Triazole-Resistant *Aspergillus fumigatus* Clinical Isolates

**DOI:** 10.3390/jof6040227

**Published:** 2020-10-16

**Authors:** Agustin Resendiz-Sharpe, Margriet W. J. Hokken, Toine Mercier, Rita Merckx, Kamiel Verhagen, Lisa Dewitte, Willem J. G. Melchers, Paul E. Verweij, Johan Maertens, Katrien Lagrou

**Affiliations:** 1 Department of Microbiology, Immunology and Transplantation, Laboratory of Clinical Bacteriology and Mycology, KU Leuven, 3000 Leuven, Belgium; agustin.resendizsharpe@kuleuven.be (A.R.-S.); toine.mercier@uzleuven.be (T.M.); rita.merckx@kuleuven.be (R.M.); lisa.dewitte@student.kuleuven.be (L.D.); johan.maertens@uzleuven.be (J.M.); 2 Department of Medical Microbiology, Radboud University Nijmegen Medical Center, Radboud Institute for Molecular Life Sciences, 6525 Nijmegen, The Netherlands; margriet.hokken@radboudumc.nl (M.W.J.H.); kamielverhagen@hotmail.nl (K.V.); Willem.Melchers@radboudumc.nl (W.J.G.M.); Paul.Verweij@radboudumc.nl (P.E.V.); 3 Department of Hematology, University Hospitals Leuven, 3000 Leuven, Belgium; 4 Center of Expertise in Mycology, Radboudumc/CWZ, 6525 Nijmegen, The Netherlands; 5 Department of Laboratory Medicine and National Reference Center for Mycosis, Excellence Center for Medical Mycology (ECMM), University Hospitals Leuven, 3000 Leuven, Belgium

**Keywords:** *Aspergillus fumigatus*, triazole-resistance, *hmg1* gene

## Abstract

Recently, mutations in the *3-hydroxy-3-methylglutaryl-coenzyme-A-reductase*-encoding gene (*hmg1*), a gene involved in ergosterol production, were associated with triazole-resistance in *Aspergillus fumigatus*. In this study, we determined the prevalence and characteristics of *hmg1* mutations in a collection of clinical triazole-resistant *A. fumigatus* isolates collected during 2001–2019 from two international mycology reference centers: the Belgian National Reference Center for Mycosis and the Center of Expertise in Mycology Radboudumc/CWZ. Clinical isolates with and without *cyp51A* gene mutations and randomly selected wild-type (WT) controls were included. Isolates were characterized by in vitro susceptibility testing, *cyp51A* and *hmg1* sequencing, and short tandem repeat typing. Available clinical records were analyzed for previous triazole exposure. In 23 isolates (24%) of the 95 triazole-resistant *A. fumigatus* isolates, *hmg1* gene mutations were observed; including 5/23 (22%) isolates without *cyp51A* gene mutations and 18/72 (25%) with *cyp51A* mutations. Four previously described *hmg1* gene mutations (E105K, G307R/D, G466V, and S541G) and two novel mutations (W273S and L304P) were found; 4/23 (17%) in the sterol-sensing-domain region. No triazole-antifungal exposure was reported in 75% (9/12) of patients harboring an isolate with *hmg1* gene mutations. Three of 39 WT isolates (8%) contained a *hmg1* gene mutation; E105K (2-isolates) and S541G. *Hmg1* gene mutations were predominantly found in *A. fumigatus* with *cyp51A* mutations with voriconazole MICs ≥ 8 mg/L.

## 1. Introduction

The triazole antifungal agents are recommended for prophylaxis (posaconazole) and first-line treatment (voriconazole and isavuconazole) of Aspergillus related-diseases [1,2]. However, the preferred use of triazoles is being threatened by reports of increasing resistance to these antifungals worldwide [3]. The most commonly reported triazole-resistance associated mechanisms in *Aspergillus fumigatus (A. fumigatus)* are genetic alterations of the *cyp51A* gene [4]. Triazole antifungals enter the active site of the Cyp51A enzyme (sterol-demethylase) blocking the access of lanosterol and its conversion to ergosterol. This leads the accumulation of toxic sterol intermediates and ergosterol depletion causing fungal cell growth inhibition and death [5]. Structural modifications in this protein due to amino acid substitutions can interrupt the access and binding of these compounds impeding their effects [5].

Nonetheless, triazole-resistant isolates without *cyp51A* mutations have also been reported in more than 50% of strains in some collections [6,7].

Recently, mutations in the *3-hydroxy-3-methylglutaryl-coenzyme A (HMG-CoA) reductase*-encoding gene (*hmg1*), were described and associated with triazole-resistance in a number of clinical *A. fumigatus* isolates [8,9,10,11,12]. Like the *cyp51A* gene, the *hmg1* gene is involved in the ergosterol production pathway [13]. HMG-CoA reductase (Hmg1) initiates ergosterol biosynthesis by reduction of HMG-CoA to mevalonic-acid and can negatively regulate its own enzymatic effects by interaction of its sterol-sensing domain region (SSD) with sterols [14,15]. Introduction of a SSD *hmg1* gene mutation (F262del, S305P, or I412S) into the *hmg1* locus of a triazole-susceptible laboratory wild-type (WT) *A. fumigatus* strain, resulted in reduction of triazole susceptibility and accumulation of ergosterol precursors without modifying *cyp51A* gene expression [9]. Replacement of these substitutions to WT *hmg1* restored susceptibility, relating these mutations to triazole-resistance. As observed by Hagiwara et al., mutations in this region may impair the inhibitory signals that initiate the degradation of Hmg1 leading to ergosterol accumulation and increasing the amount of triazole antifungal required to inhibit fungal growth [8]. The prevalence of *hmg1* gene mutations and their role in respect to triazole-resistance in *A. fumigatus* is not well known. In this study, we determined the prevalence of mutations in the *hmg1* gene in a large collection of triazole-resistant *A. fumigatus* isolates with and without *cyp51A* gene mutations from two international mycology reference centers.

## 2. Materials and Methods

### 2.1. Aspergillus fumigatus Clinical Isolates and Triazole-Resistance Determination

Clinical *A. fumigatus* isolates received at the National Reference Center for Mycosis UZ Leuven in Belgium (between 2012 and July 2019) and at the Center of Expertise in Mycology Radboudumc/CWZ in the Netherlands (between 2001 and 2017) were screened for inclusion in this study. The inclusion criteria were as follows: (a) triazole-resistant isolates with WT-*cyp51A* gene; (b) triazole-resistant isolates with *cyp51A* gene mutations subdivided in three subgroups TR_34_/L98H, TR_46_/Y121F/T289A, and others (maximum 15 isolates per year from each subgroup); (c) 39 randomly selected triazole-susceptible WT isolates (based on an *hmg1* mutation prevalence of 24%, power 0.9, alpha 0.05); and maximum one isolate per patient within a four-month period. Triazole-resistance screening of *A. fumigatus* isolates followed by susceptibility determination (EUCAST broth microdilution reference method) of suspected triazole-resistant isolates were performed as previously described [16]. Isolates were designated as triazole-resistant if at least one minimal inhibitory concentration value (MIC) was above the established EUCAST resistance clinical breakpoints for *A. fumigatus* (voriconazole >2, itraconazole >2, posaconazole > 0.25, mg/L) at the time of isolates selection [17]. The presence of mutations within the *cyp51A* gene and its promotor region was assessed by sequencing as reported before [18]. 

### 2.2. hmg1 Gene Sequencing

Amplified DNA from selected *A. fumigatus* isolates was sequenced using specifically designed primers (Table S1) and the BigDye-Terminator-v3.1 cycle-sequencing kit (Applied-Biosystems, Lithuania). Reaction products were purified (DyeEX2.0-Kit; Qiagen, Germany), dried, and reconstituted in 20 μL of HiDi Formamide (Applied Biosystems, UK) according to manufacturer and run on an ABI3730xl Genetic Analyzer (Applied-Biosystems, USA). Obtained sequences were aligned to create a consensus sequence using the CLC-Genomics-Workbench software (CLC-bio, Denmark). Sequence variant detection was performed by comparison of each isolate consensus sequence to the *A. fumigatus hmg1* gene reference sequence (FungiDB:AFUB_020770_A 1163).

### 2.3. Genotyping

Genetic relatedness of isolates with *hmg1* gene mutations and without *hmg1* gene was determined using two separate multiplex PCRs specific for *A. fumigatus* (short tandem repeats) of three trinucleotide loci (M3_*STRAf*3A-C primers) and three tetranucleotide loci (M4_*STRAf*4A-C primers), respectively, as described by de Valk HA. et al. [19] (Applied Biosystems, UK; Table S1). Fragments (4 μL of cleaned PCR product combined with HiDi Formamide and GeneScan 500 LIZ size standard; Applied Biosystems, UK) were detected on a 96-capillary array ABI3730xl Genetic Analyzer (Applied Biosystems, USA). Number of repeats were determined using the Peak Scanner Software v1.0 (Applied Biosystems). Isolates genetic distance (Euclidean method) and hierarchical relationship (Ward’s minimum variance linkage clustering algorithm method; ggplot2 package) were performed with the statistical computing and graphics software R (version 4.0.2) [20].

### 2.4. Antifungal Exposure and Clinical Data Management

Patient electronic records were retrospectively consulted to check for exposure to any antifungal either as treatment or as prophylaxis. Use of any antifungal was recorded from 30-days before the isolate was cultured. Antifungal exposure in patients with multiple isolates was determined based on culture of the first triazole-resistant isolate. This study was performed in accordance to each center ethical regulations.

## 3. Results

### 3.1. hmg1 Gene Mutations Are Prevalent among Triazole-Resistant Aspergillus fumigatus Isolates

**Figure 1:**
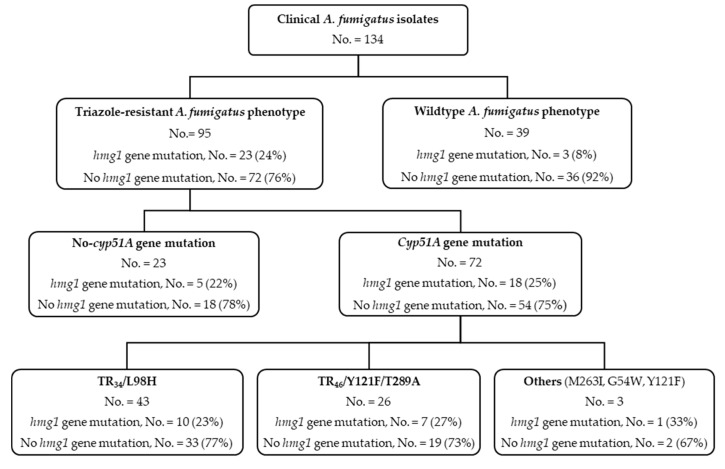
*hmg1* gene mutations in triazole-susceptible and triazole-resistant *Aspergillus fumigatus* clinical isolates.

**Figure 2:**
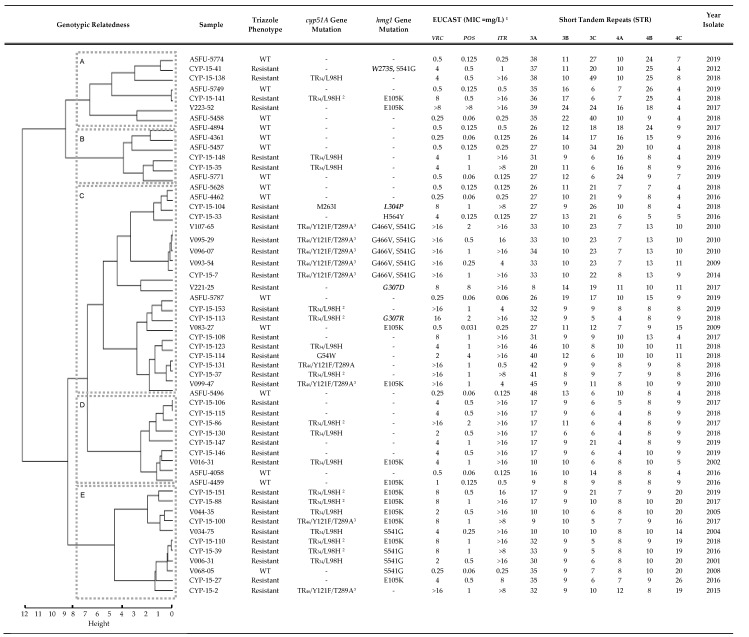
Characteristics and Genetic Relatedness of *Aspergillus fumigatus* Clinical Isolates Harboring *hmg1* Gene Mutations and of Selected Isolates without *hmg1* Gene Mutations. ^1^ EUCAST broth microdilution reference method for filamentous fungi. ^2^ Atypical TR_34_/L98H (elevated) MIC values: itraconazole ≥ 4, voriconazole ≥ 8, posaconazole ≥ 0.5 mg/L. ^3^ Atypical TR_46_/Y121F/T289A phenotype (elevated) MIC values: voriconazole ≥ 4, itraconazole ≥ 4, posaconazole ≥ 0.25 mg/L. Amino-acid variances located in the sterol-sensing domain (SSD) are depicted in bold and Italic (SSD, 242-415, genome AFUB_020770). Abbreviations: MIC (Minimal inhibitory concentration), VRC (voriconazole), POS (posaconazole), ITR (itraconazole). Not detected = “-”.

In total 95 triazole-resistant and 39 triazole-susceptible *A. fumigatus* clinical isolates, from 92 patients (two patients with 3 (cyp-15-106, 123, 138) and 2 (cyp-15-141,146) genetically unrelated triazole-resistant isolates, respectively), were evaluated (Figure 1 and Figure 2). *Hmg1* gene mutations were observed in 24% (23/95 isolates, *p* = 0.0310) of triazole-resistant isolates (Figure 1 and Figure 2), 4.2% (4/95) in the SSD region (amino acids 242–415; *hmg1* genome reference AFUB_020770). Among the triazole-resistant isolates, *hmg1* mutations were found in 22% (5/23) of isolates with no-*cyp51A* gene mutations and 25% (18/72) of isolates with *cyp51A* gene mutations; 10 of 43 (23%) isolates in the TR_34_/L98H subgroup, 7 of 26 (27%) in the TR_46_/Y121F/T289A subgroup, and one of 3 (33%) in the group with other mutations. *Hmg1* gene mutations were found in three (8%) of the 39 susceptible WT *A. fumigatus* clinical isolates; harboring the previously described amino acid substitutions E105K (two isolates) and S541G (one isolate) (Figure 2). Phenotypic and genetic characteristics of *A. fumigatus* isolates without *hmg1* gene mutations are depicted in Figure 2 and Table S2 as well.

Genetic relatedness analysis of isolates with *hmg1* gene mutations (23 triazole-resistant and 3 susceptible isolates) and without *hmg1* gene mutations (16 triazole-resistant and 12 WT isolates) showed five clusters (A–E, Figure 2) occurring at the same horizontal distance with an average Euclidean distance of 3.2, varying among isolates from 0.0 to 7.08. Two of the analyzed isolates harboring TR_46_/Y121F/T289A *cyp51A* gene and G466V, S541G *hmg1* gene mutations (V107-65 and V095-29) and two triazole-resistant isolates with no *cyp51A* or *hmg1* gene mutations (CYP-15-106 and CYP-15-115) were genetically identical; Euclidean distance 0.0). Among *hmg1* triazole-resistant isolates, 3 out of 23 (13%) were clustered together in clade A, none in clade B, 10 in clade C (44%), one in clade D (4%) and 9 in clade E (39%). In clade E, 60% (6/10) of *hmg1* triazole-resistant isolates harboring the amino acid substitution E105K and all triazole-resistant isolates (3/3) with the S541G one were found. Isolates with *hmg1* gene mutations in the SSD were mostly found in clade C (3/4, 75%) in addition to all triazole-resistant isolates harboring the G466V and S541G amino acid substitutions (5/5, 100%). WT isolates with the E105K were clustered in clade C and D, and the S541G in clade E where most triazole-resistant isolates harboring this mutation are situated. *Hmg1* gene mutations were found in isolates from our collection since 2001, our starting study year.

### 3.2. Triazole Phenotypes in Isolates with Combined cyp51A Gene and hmg1 Gene Mutations

The TR_34_/L98H associated resistant-phenotype consists of elevated itraconazole MICs (>4 mg/L), and variable voriconazole and posaconazole MICs [21]. We classified two TR_34_/L98H resistance phenotypes: typical-TR_34_/L98H phenotype (29/43 isolates) characterized by itraconazole MIC ≥ 4 mg/L, voriconazole MIC ≤ 4 mg/L, and variable posaconazole MIC; and atypical-TR_34_/L98H phenotype (14/43 isolates) characterized by itraconazole MIC ≥ 4 mg/L, voriconazole MIC ≥ 8 mg/L, and posaconazole MIC ≥ 0.5 mg/L. Isolates harboring *hmg1* gene mutations were found mostly in the isolates with an atypical-TR_34_/L98H phenotype (6 of 14 (43%; one in the SSD [7%]) versus 4 of 29 (14%) typical-TR_34_/L98H phenotype isolates; *p* = 0.0549).

TR_46_/Y121F/T289A mutations are typically related to high-level voriconazole resistance (MIC > 4 mg/L), and variable itraconazole and posaconazole susceptibility [22]. TR_46_/Y121F/T289A isolates could also be categorized according to their MIC profile into typical-TR_46_/Y121F/T289A phenotype (voriconazole MIC ≥ 4 mg/L, itraconazole MIC ≤ 2 mg/L, and variable posaconazole MICs; 13/26 isolates) and atypical-TR_46_/Y121F/T289A phenotype (voriconazole MIC ≥ 4, itraconazole MIC ≥ 4, and posaconazole MIC ≥ 0.25 mg/L; 13/26 isolates) groups. *Hmg1* mutations were only observed in isolates with an atypical azole phenotype with a prevalence of 54% (7 of 13 atypical-TR_46_/Y121F/T289A phenotype, *p* = 0.0052). Among the other *cyp51A* gene group, one isolate with the M263I mutation harbored a mutation in the *hmg1* gene located in the SSD (1 of 3; 33%).

### 3.3. Previous Exposure to Triazole Antifungals Is Uncommon among Patients Harboring Isolates with hmg1 Gene Mutations

Data about exposure to triazole antifungals could be retrieved for 51 of 92 patients with triazole-resistant isolates (55%). Exposure to triazole antifungals was observed in 25% (3/12) of patients with *hmg1* gene mutation compared to 75% (9/12) in those without triazole-antifungal exposure (Table 1). No previous triazole exposure was observed in the two patients harboring a WT-isolate with the E105K *hmg1* gene mutation.

## 4. Discussion

We report a prevalence of 24% *hmg1* gene mutations (23/95) in general and of 4.2% in the SSD region (4/95) in our collection of triazole-resistant clinical *A. fumigatus* isolates. Our reported prevalence is lower than observed in previous reports. Rybak et al. reported *hmg1* gene mutations in 52% (11/21) of their clinical triazole-resistant *A. fumigatus* isolates, while a prevalence of 45.5% (5/11) was found in isolates from Manchester, UK [9]; however, the number of cases in these studies were small and, as only few *hmg1* gene mutations have been related to resistance in *A. fumigatus* and not all might have the potential to confer resistance we cannot draw a firm conclusion. *hmg1* gene mutations could be accountable as the underlying mechanism conferring triazole-resistance in 5 of 23 (22%; two in the SSD [9%]) isolates without *cyp51A* gene mutations, but other mechanisms conferring triazole-resistance cannot be excluded. The MICs of these isolates were above the clinical breakpoint for voriconazole of 2 mg/L (MIC ≥ 4 mg/L), while a variable phenotype was observed for posaconazole (MIC 0.125 to >8 mg/L) and itraconazole (MIC 0.125 to >8 mg/L).

Mutations in the *hmg1* gene were also observed among triazole-resistant isolates with known azole-resistant related *cyp51A* gene mutations. Interestingly, *hmg1* gene mutations were detected mostly in isolates harboring tandem repeat mutations categorized as atypical resistance phenotype, with a prevalence of 43% (6/14) in atypical-TR_34_/L98H isolates group and 54% (7/13) in atypical-TR_46_/Y121F/T289A compared to 14% (4/29) and 0% (0/13) in typical-TR_34_/L98H and -TR_46_/Y121F/T289A ones, respectively. It may appear that genetic variances in the *hmg1* gene in isolates harboring known associated triazole-resistant *cyp51A* gene mutations further modulate triazole-resistance elevating MIC values from conventional expected ones as reported in other *non-cyp51A* gene mediated mutations [23]. Additional investigation is required to determine if this is indeed the case for these *cyp51A* gene mutations and the mechanisms involved.

**Figure 3:**
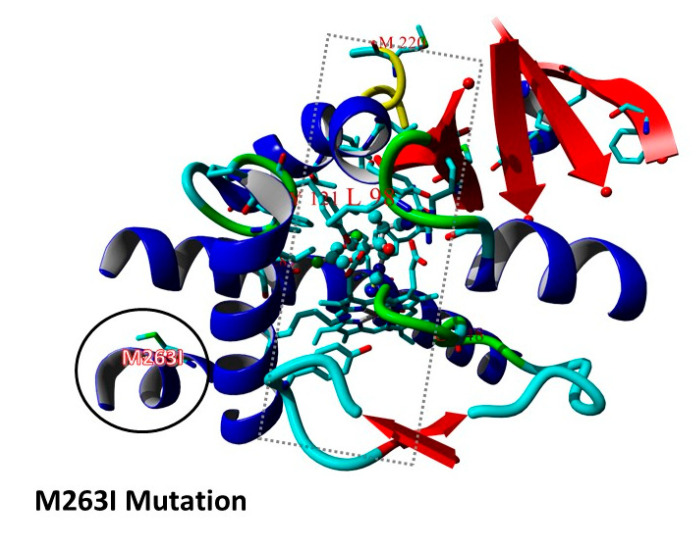
Representative schematic of the M263I mutation in the *Aspergillus fumigatus* Cyp51A homology protein model. Predicted location of the M263I *cyp51A* gene mutation in an optimized static *Aspergillus fumigatus* Cyp51A molecular docking and molecular dynamics homology protein model (MM/MD) of *Aspergillus fumigatus*. Rectangle (dotted) contains the predicted triazole antifungals binding region and commonly associated region of known resistance mutations. Circle (plain) contains the predicted location of mutation M263I.

Genetic variances in the SSD of the *hmg1* gene have been associated with triazole-resistance in *A. fumigatus* [8,9]. Among our triazole-resistant isolates, 4 out of 95 (4.2%) presented amino-acid variances in this region, two at novel locations W273S and L304P, and two in the previously described G307 position (Table 2), with an amino acid change from glycine to arginine (G307R) or to aspartic acid (G307D). The *hmg1* amino acid substitution L304P was found in an isolate from the other *cyp51A* group with the M263I mutation. Rybak et al., reported two isolates harboring the M263I mutation in which substitution of their *cyp51A* gene to a WT one, did not restore susceptibility [9]. Furthermore, incorporation of the position of the M263I into a protein homology model of *Aspergillus fumigatus* [5] (Figure 3) showed that this mutation is positioned outside on the protein surface and not in the well-known associated triazole-resistance protein regions (triazole-antifungals entrance or ion-exchange channels) [5].

The most prevalent *hmg1* mutation found among triazole-resistant isolates in our collection was the previously described amino-acid change E105K [9] (10/23 isolates, 44%). E105K is located at the beginning of the membrane bound region but not in the SSD region and its exact role in triazole-resistance is not known. E105K was mostly found in isolates (7/10, 70%) with an atypical resistance phenotype (MIC: ≥4 mg/L itraconazole, ≥8 mg/L voriconazole). Our second and third most prevalent mutations were G466V, S541G (5/23, 22%) and S541G (3/23, 13%) [9], also located outside the SSD region. Most isolates harboring these mutations were likewise classified as having an atypical resistance phenotype; G466V, S541G (5/5, 100%), and S541G (2/3, 67%). As is the case for the amino acid variances in the TR_46_/Y121F/T289A *cyp51A* mutation [22], certain *hmg1* gene amino acid substitutions may to some extent synergize the effects of other resistance mechanisms that confer triazole-resistance (*cyp51A* or other *hmg1* mutations).

Within our study, the E105K and the S541G mutation were also found in two (5%) and one (3%), respectively, of 39 isolates with a triazole WT phenotype. The S541G and H564Y *hmg1* mutations located likewise outside the SSD, have also been reported in non-resistant isolates [8,11]. This a major issue, as it raises the probability that these mutations located outside the SSD most likely do not confer resistance and that other mechanisms are responsible for the observed resistant phenotypes. We cannot discard the possibility that compensatory mutations, which could modulate azole susceptibility to WT MICs, exist in the non-resistant isolates harboring these mutations. Triazole-resistant *A. fumigatus* isolates harboring tandem repeat mutations, such as TR_34_/L98H, are known to be closely related [24]. Our genetic relatedness analysis indicates that 60% (6/10) of E105K isolates (in addition to one E105K WT isolate) and all (3/3) S541G triazole-resistant isolates (including the WT isolate harboring this mutation) are closely related (clade E), suggesting the emergence of variants from a local common ancestor which decreases the likelihood of being a commonly distributed. The possibility that these mutations might confer other beneficial effects in regard to survival or fitness should also be considered. Recombinant experiments are required to determine whether E105K and S541G mutations, and others found outside the SSD, play a role in conferring triazole-resistance in *A. fumigatus* or are common polymorphisms of the Hmg1 locus.

Recently, Nakano et al. [25] reported the presence of *hmg1* gene mutations in 14 triazole-resistant *A. fumigatus* environmental samples from Japan (1/14) and the Netherlands (13/14) harboring the E105K and the S541G (AfuB_020770 genome) amino acid substitutions (voriconazole MICs 8 mg/L, itraconazole MICs 0.5 → 8 mg/L); all located outside the SSD. In our study, we observed that the majority of *A. fumigatus* isolates with *hmg1* gene mutations (75%, 9/12; 1 in the SSD region) were isolated from patients who had no previous triazole-antifungal exposure, further inferring that these mutations might have been previously selected in the environment, as is the case in most *cyp51A* gene mutation infections in patients [3], but due to low number of cases, results were not significant and should be interpreted with caution. Larger environmental *hmg1* triazole-resistance screenings should be undertaken to investigate this more in depth. Our study has some limitations. First, the number of triazole-resistant isolates with no- and others-*cyp51A* gene mutations in our collections were low, but to the best of our knowledge, this is the largest collection tested so far. Second, clinical data could not be retrieved from all patients.

In this study we systematically analyzed the prevalence of *hmg1* gene mutations in a collection of *A. fumigatus* clinical isolates in two international mycology reference centers. In addition to isolates without *cyp*51A mutations, *hmg1* gene mutations were also detected in isolates with *cyp*51A mutations mostly in isolates with a voriconazole MIC of ≥4 mg/L and a posaconazole MIC of ≥0.25 mg/L. Further studies are needed to unravel the mechanistic role and clinical implications of *hmg1* mutations.

## Figures and Tables

**Table 1:** Exposure to Triazole Antifungals in Patients with Triazole-Resistant *Aspergillus fumigatus* Isolates According to *hmg1* Gene Mutations.

Triazole-Resistant *A. fumigatus* Cases	Triazole Antifungal Exposure ^1^	No Triazole Antifungal Exposure ^2^	Total Cases
*Hmg1* gene mutation, no. (%)	3 (25)	9 (75)	12 (100)
No *hmg1* gene mutation, no. (%)	7 (18)	32 (82)	39 (100)

^1^ Defined as use of any triazole antifungal 30 days before the isolate was cultured (itraconazole, voriconazole, posaconazole, isavuconazole). ^2^ No or other antifungal exposure (echinocandins, liposomal amphotericin B, olorofim). Fisher’s exact test, *p* = 0.6822.

**Table 2:** Reported Clinical *Aspergillus fumigatus* Isolates with Mutations in the *hmg1* Gene.

*hmg1* Gene Mutation (Location)		Number of Reported Clinical *A. fumigatus* Isolates *					
	Hagiwara et al.*	Rybak et al.*	Sharma et al.*	Chi-Jung et al.*	Takeda et al. *	This study	Total
E105K		1				12	13
***S269F, F390Y***			1				1
***S269P****,* H564Y				4 ^2^			4
**Y250H**		1					1
***F261del***	1						1
***F262del***		1					1
***F262del****,* H564Y				2 ^2^			2
***S269F***	18 ^2^						18
***S269Y***	1						1
***L273F***					1		1
***W273S****,* S541G						1	1
***L304P***						1	1
***S305P***		1			1		2
***S305P****,* V995I		1					1
***G307D***		1				1	2
***G307R***						1	1
***IP309L***		1					1
***F390Y***	1						1
***I412S***		2					2
***I412T***		1					1
***L413P***		1					1
G466V, S541G		1				5	6
S541G ^1^						4	4
H564Y ^1^	1			3		1	5
V995I		1					1

Only relevant mutations identified when compared to reference genome AFUB_020770 (GenBank: EDP54027.1) are mentioned in this table. Amino-acid variances located in the sterol-sensing domain region (SSD; 242-415) are in bold and Italic. ^1^ Amino-acid variance previously reported also in susceptible *A. fumigatus* isolates. ^2^ All isolates belonged to the same patient. * References: Hagiwara et al. [8]., Rybak et al. [9]., Sharma et al. [10]., Chi-Jung et al. [11], Takeda et al. [12].

## Supplementary Materials

The following are available online at https://www.mdpi.com/2309-608X/6/4/227/s1, Table S1. *Aspergillus fumigatus hmg1* gene and genotyping primer sequences, Table S2. Characteristics of *Aspergillus fumigatus* clinical isolates without *hmg1* gene mutations.
