# Supplementary material for: Hmg1 Gene Mutation Prevalence in Triazole-Resistant Aspergillus fumigatus Clinical Isolates
Source: J Fungi (Basel). 2020 Oct 16;6(4):227. doi: 10.3390/jof6040227 (PMC7711918; doi:10.3390/jof6040227)
Supplement: Supplementary file 1 [file jof-06-00227-s001.pdf]

Supplementary Materials:

Table S1. –*Aspergillus fumigatus hmg1* gene and genotyping primer sequences.

Name	Template strand	Sequence (5'- 3')
***hmg1* gene** [1]		
**Belgian center**		
hmg1_SQNCBI_F1*	Forward primer	CATTCCTGAGATTTCTCAGCATCGA
hmg1_SQNCBI_R1	Reverse primer	GTCCTGGCTCGCTAAACTGT
hmg1_SQ2F	Forward primer	ACAGTTTAGCGAGCCAGGAC
hmg1_SQNCBI_R2	Reverse primer	AATGGCGATCTCGATGCAGT
hmg1_SQNCBI_F3	Forward primer	TGCATTCACCTTGCCGTTTG
hmg1_SQNCBI_R3	Reverse primer	AAGAGTGATGTGGCGCTTGA
hmg1_SQNCBI_F6	Forward primer	GCGGCGTGGATCTTGTTTTT
hmg1_SQNCBI_R6	Reverse primer	AAAGGGTCAATGGGCGTAGG
hmg1_SQNCBI_R7	Reverse primer	CTGTGATAGCGGAAGTGGCA
hmg1_SQNCBI_F8	Forward primer	GACCCTTTCAAGGTCGCAGA
hmg1_SQNCBI_R8	Reverse primer	GTGTCGGGGTTCAAGTCGAT
hmg1_SQNCBI_F9	Forward primer	ACGGTTGTGACAGTCATCCC
hmg1_SQNCBI_F10	Forward primer	ATCGACTTGAACCCCGACAC
hmg1_SQNCBI_R10	Reverse primer	GGTATTGAGCTCGACCAGGG
hmg1_SQNCBI_F11	Forward primer	GCTCTCGCTGGCACTTATCT
hmg1_SQNCBI_R11*	Reverse primer	CCATGTGTATTTCGGACAGCCAGC
**Dutch center**		
hmg1_PCR_Fw*	Forward primer	CTCCTGCTCGCCATTTTG
hmg1_Fw_int1	Forward primer	AGGTGGAGGACAGTTTAGC
hmg1_Fw_int2	Forward primer	CAATTTCTGAAAGCGGTG
hmg1_Fw_int3	Forward primer	CTAACTTCTGGTTGGCTG
hmg1_Fw_int4	Forward primer	GCGACAATTCTGTTTTCTC
hmg1_Fw_int5	Forward primer	CTACTGTCTCGTGTCTCTAATG
hmg1_Fw_int6	Forward primer	CTCAAGAGTATCGAAGATCC
hmg1_Fw_int7	Forward primer	CCACTTCCGCTATCACAG
hmg1_Fw_int8	Forward primer	CTTGTGTCGGTTTCCCTAC
hmg1_Fw_int9	Forward primer	GTCGAGCTCAATACCAGTAAG
hmg1_Fw_int10	Forward primer	GATATGCTAGGTGTCCGTG
hmg1_PCR_Rv*	Reverse primer	CGCGAACTCCCGAATAGTA
STRAf analysis		
M3_STRAf3A_F	Forward primer	FAM-GCTTCGTAGAGCGGAATCAC
M3_STRAf3A_R	Reverse primer	GTACCGCTGCAAAGGACAGT
M3_STRAf3B_F	Forward primer	HEX/VIC[2]-CAACTTGGTGTCAGCGAAGA
M3_STRAf3B_R	Reverse primer	GAGGTACCACAACACAGCACA
M3_STRAf3C_F	Forward primer	TED/NED[2]-GGTTACATGGCTTGGAGCAT
M3_STRAf3C_R	Reverse primer	GTACACAAAGGGTGGGATGG
M4_STRAf4A_F	Forward primer	FAM-TTGTTGGCCGCTTTTACTTC
M4_STRAf4A_R	Reverse primer	GACCCAGCGCCTATAAATCA
M4_STRAf4B_F	Forward primer	HEX/VIC[2]-CGTAGTGACCTGAGCCTTCA
M4_STRAf4B_R	Reverse primer	GGAAGGCTGTACCGTCAATCT
M4_STRAf4C_F	Forward primer	TED/NED[2]-CATATTGGGAAACCCACTCG
M4_STRAf4C_R	Reverse primer	ACCAACCCATCCAATTCGTAA

[1] Target gene: Hydroxymethylglutaryl-CoA reductase genomic sequence (hmg1) of A. fumigatus (FungiDB Gene ID: AFUB_020770, A1163).
[2] Fluorescent dyes used at the Belgian center
* Primers used for PCR reaction.

Table S2. - Characteristics of *Aspergillus fumigatus* clinical isolates without *hmg1* gene mutations.

Clinical *A. fumigatus* isolates	*hmg1* gene mutation (amino-acid substitution)	EUCAST (MIC =mg/L) [1]		
		Voriconazole	Posaconazole	Itraconazole
No-cyp51A gene associated-resistance mutations				
CYP-15-18	-*	8	0.5	1
CYP-15-75	-	4	1	8
CYP-15-93	-	4	1	>16
CYP-15-106	-	4	0.5	>16
CYP-15-108	-	8	1	>16
CYP-15-109	-	>8	1	2
CYP-15-115	-	4	0.5	>16
CYP-15-117	-	0.25	0.5	>16
CYP-15-146	-	4	0.5	>16
CYP-15-147	-	4	1	>16
V156-11	-	4	0.5	>16
V215-17	-	4	1	>16
V217-27	-	2	0.25	4
V220-62	-	>8	0.25	1
V220-73	-	8	0.5	>16
V221-53	-	2	0.5	>16
V222-68	-	4	1	>16
V224-65	-	1	1	>16
Typical TR_{34}/L98H phenotype [2]				
CYP-15-24	-	4	1	>8
CYP-15-35	-	4	1	>8
CYP-15-44	-	2	1	8
CYP-15-94	-	4	0.5	>8
CYP-15-103	-	4	1	>8
CYP-15-112	-	4	0.5	>16
CYP-15-120	-	2	0.5	>16
CYP-15-121	-	2	0.5	>16
CYP-15-123	-	4	1	>16
CYP-15-130	-	2	0.5	>16
CYP-15-132	-	2	0.25	4
CYP-15-135	-	4	0.5	>16
CYP-15-138	-	4	0.5	>16
CYP-15-143	-	4	0.5	>16

Continuation Table S2

CYP-15-144	-	2	0.25	4
CYP-15-148	-	4	1	>16
CYP-15-150	-	2	0.25	>16
CYP-15-152	-	2	0.25	>16
CYP-15-155	-	2	0.5	>16
CYP-15-157	-	4	0.5	>16
CYP-15-158	-	1	0.5	>16
CYP-15-160	-	4	0.5	>16
V006-59	-	4	0.5	>16
V017-07	-	2	0.5	>16
V027-28	-	4	0.25	>16
Atypical TR_{34}/L98H phenotype [3]				
CYP-15-37	-	>16	1	>8
CYP-15-73	-	8	1	>8
CYP-15-86	-	>16	2	>16
CYP-15-128	-	8	1	>16
CYP-15-153	-	>16	1	4
V041-26	-	>16	1	>16
V049-77	-	8	0.5	>16
V052-32	-	8	0.5	>16
		L		
Typical TR_{46}/Y121F/T289A phenotype [4]				
CYP-15-30	-	>8	1	1
CYP-15-57	-	>16	0.5	1
CYP-15-60	-	>16	0.5	1
CYP-15-80	-	>16	0.5	0.5
CYP-15-83	-	>16	0.5	2
CYP-15-99	-	>16	1	2
CYP-15-111	-	>16	0.5	1
CYP-15-129	-	>16	0.25	0.25
CYP-15-131	-	>16	1	0.5
CYP-15-159	-	>16	0.5	1
V092-61	-	>16	0.5	2
V094-16	-	>16	0.25	0.5
V098-33	-	>16	1	1
Atypical TR_{46}/Y121F/T289A phenotype [5]				
CYP-15-2	-	>16	1	>8
CYP-15-38	-	>16	1	>8
CYP-15-118	-	>16	0.5	8
CYP-15-156	-	>16	1	>16
CYP-15-161	-	>16	0.5	4
V096-59	-	>16	1	>16

Continuation Table S2

Other cyp51A gene associated-resistance mutations				
CYP-15-114 (G54W)	-	2	4	>16
CYP-15-162 (Y121F)	-	>16	1	2
Triazole-susceptible isolates (Wild-type)				
ASFU-4026	-*	1	0.125	0.5
ASFU-4058	-	0.5	0.06	0.125
ASFU-4361	-	0.25	0.06	0.125
ASFU-4374	-	0.25	0.06	0.125
ASFU-4462	-	0.25	0.06	0.25
ASFU-4520	-	1	0.125	0.5
ASFU-4701	-	0.25	0.125	0.06
ASFU-4894	-	0.5	0.125	0.5
ASFU-5291	-	0.06	0.25	0.125
ASFU-5457	-	0.5	0.125	0.25
ASFU-5458	-	0.25	0.06	0.25
ASFU-5496	-	0.25	0.06	0.125
ASFU-5549	-	1	0.125	1
ASFU-5628	-	0.5	0.125	0.125
ASFU-5745	-	1	0.125	0.5
ASFU-5749	-	0.5	0.125	0.5
ASFU-5771	-	0.5	0.06	0.125
ASFU-5774	-	0.5	0.125	0.25
ASFU-5779	-	0.25	0.06	0.125
ASFU-5787	-	0.25	0.06	0.06
V013-70	-	0.5	0.06	0.125
V014-76	-	0.5	0.125	0.5
V015-77	-	0.25	0.06	0.25
V037-14	-	1	0.06	0.25
V039-02	-	1	0.06	0.25
V045-05	-	1	0.06	0.25
V046-16	-	1	0.06	0.125
V047-36	-	0.5	0.031	0.125
V54-73	-	0.5	0.06	0.25
V056-06	-	0.25	0.125	0.25
V062-59	-	1	0.125	0.5
V062-81	-	1	0.06	0.5
V068-62	-	0.25	0.06	0.125
V069-67	-	0.25	0.031	0.125
V082-62	-	0.5	<0.016	0.125
V083-54	-	0.5	0.06	0.25

[1] EUCAST broth microdilution reference method for filamentous fungi. * Not detected = " - ".

[2] Typical TR_{34}/L98H MIC values: itraconazole ≥ 4, voriconazole ≤ 4 mg/L, posaconazole variable (0.25-1 mg/L).

[3] Atypical TR_{34}/L98H (elevated) MIC values: itraconazole ≥ 4, voriconazole ≥ 8, posaconazole ≥ 0.5 mg/L.

[4] Typical TR_{46}/Y121F/T289A MIC values: voriconazole ≥ 4, itraconazole ≤ 2 mg/L, posaconazole variable (0.25-1) mg/L.

[5] Atypical TR_{46}/Y121F/T289A (elevated) MIC values: voriconazole ≥ 4, itraconazole ≥ 4, posaconazole ≥ 0.5 mg/L.
